# The global challenges and opportunities in the practice of rheumatology: White paper by the World Forum on Rheumatic and Musculoskeletal Diseases

**DOI:** 10.1007/s10067-014-2841-6

**Published:** 2014-12-14

**Authors:** Mustafa Al Maini, Femi Adelowo, Jamal Al Saleh, Yousef Al Weshahi, Gerd-Rüdiger Burmester, Maurizio Cutolo, Joseph Flood, Lyn March, Heather McDonald-Blumer, Kevin Pile, Carlos Pineda, Carter Thorne, Tore K. Kvien

**Affiliations:** 1 Mafraq Hospital, Abu Dhabi, United Arab Emirates; 2 Lagos State University College of Medicine, Ikeja, Nigeria; 3 Dubai Hospital, Dubai, United Arab Emirates; 4 Oman Medical Specialty Board, Muscat, Sultanate of Oman; 5 Department of Rheumatology and Clinical Immunology, Charité University Medicine Berlin, Humboldt University, Berlin, Germany; 6 Division of Rheumatology, Department of Internal Medicine, University of Genova, Genova, Italy; 7 Ohio State University College of Medicine and Public Health and Columbus Arthritis Center, Columbus, OH USA; 8 University of Sydney Institute of Bone and Joint Research and Department of Rheumatology, Royal North Shore Hospital, St Leonards, Australia; 9 Department of Medicine, University of Toronto, Toronto, Canada; 10 University of Western Sydney, Sydney, NSW Australia; 11 Instituto Nacional de Rehabilitación, Mexico City, Mexico; 12 University of Toronto and Southlake Regional Health Centre, Newmarket, ON Canada; 13 Department of Rheumatology, Diakonhjemmet Hospital, Oslo, Norway; 14 WFRMD, PO BOX 77893, Abu Dhabi, United Arab Emirates

**Keywords:** Clinical trials, Diagnostic tests, Epidemiology, Medical education, Public health, Rheumatic diseases

## Abstract

Rheumatic and musculoskeletal diseases (RMDs) represent a multitude of degenerative, inflammatory and auto-immune conditions affecting millions of people worldwide. Persons with these diseases may potentially experience severe chronic pain, joint damage, increasing disability and even death. With an increasingly ageing population, the prevalence and burden of RMDs are predicted to increase, placing greater demands on the global practice of rheumatology and related healthcare budgets. Effective treatment of RMDs currently faces a number of challenges in both the developed and developing world, and individual countries may face more specific local challenges. However, limited understanding of the burden of RMDs amongst public health professionals and policy-makers means that these diseases are often not considered a public health priority. The objective of this review is to increase awareness of the RMDs and to identify opportunities to address RMD challenges on both a local and global scale. On 26 September 2014, rheumatology experts from five different continents met at the World Forum on Rheumatic and Musculoskeletal Diseases (WFRMD) to discuss and identify some key challenges for the RMDs community today. The outcomes are presented in this review, focusing on access to rheumatology services, diagnostics and therapies, rheumatology education and training and on clinical trials, as well as investigator-initiated and epidemiological research. The long-term vision of the WFRMD is to increase perception of the RMDs as a major burden to society and to explore potential opportunities to improve global and local RMD care.

**Electronic supplementary material:**

The online version of this article (doi:10.1007/s10067-014-2841-6) contains supplementary material, which is available to authorized users.

## Introduction

**Fig. 1:**
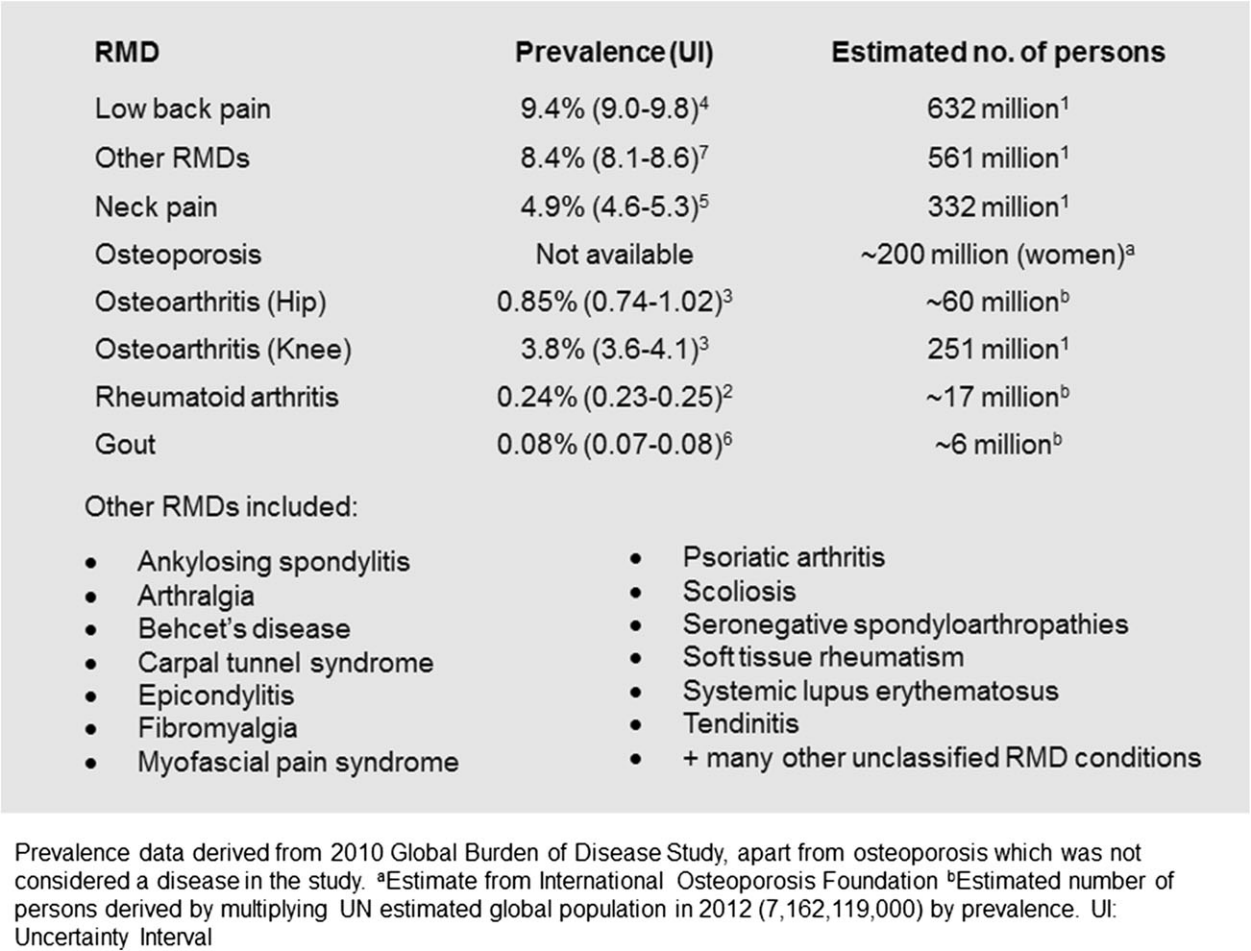
Rheumatic and musculoskeletal diseases (RMDs) and their estimated prevalence

The term rheumatic and musculoskeletal diseases (RMDs) largely encompasses over one hundred degenerative, inflammatory and auto-immune conditions which in their most advanced form are associated with severe pain, joint damage, disability and even death. In the 2010 World Health Organization (WHO) Global Burden of Disease Study, RMDs were reported to be the second leading cause of disability worldwide, as measured by years lived with disability [1]. Estimates suggest that almost 2 billion people are affected worldwide [1] imposing huge financial costs; in Europe alone, RMDs are associated with an economic burden of over €200bn per year. The global burden of individual RMDs has recently been addressed in a series of individual articles (Fig. 1) [2–7]. Nevertheless, awareness of the burden of RMDs amongst policy-makers remains limited for both paediatric and adult manifestations of diseases. With an ageing global population, the prevalence and burden of RMDs in developing and developed countries^1^ is predicted to increase, resulting in reduced quality of life and loss of work productivity, while placing a major burden on national healthcare systems [8, 7].

**Fig. 2:**
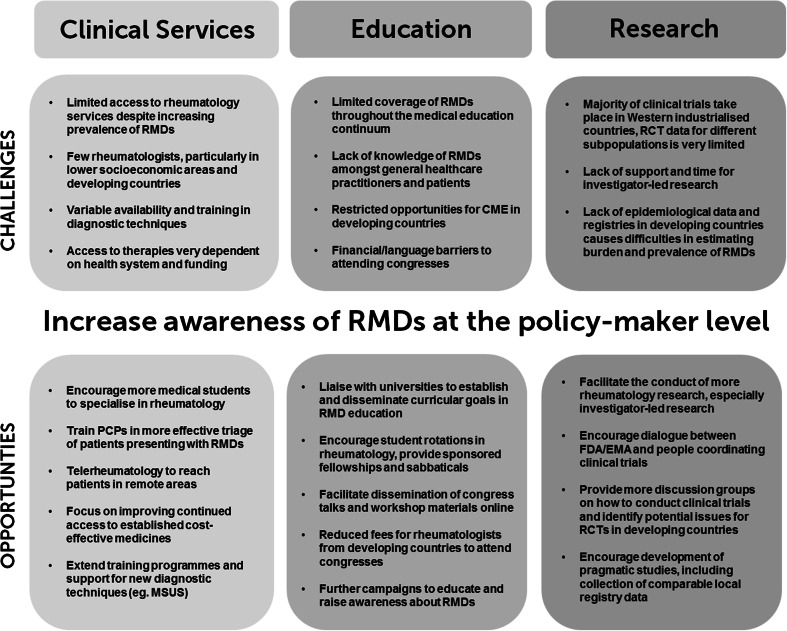
Summary of the global challenges and opportunities facing rheumatology today

Worldwide inequalities exist in access to clinical care, rheumatology training and research opportunities. However, the burden of disease is often higher in developing countries, due to limited access to clinical services and treatments [9, 10]. Increasing awareness amongst policy-makers of the health problems and economic burdens associated with RMDs, in order to prioritise the RMDs in healthcare planning, will help ensure the best possible patient outcomes. Over the past decade, the WHO has developed a global strategy for the treatment of many non-communicable diseases; however, RMDs are not mentioned [11] and are not indexed as a topic on the WHO website. Other initiatives such as the Bone and Joint Decade (BJD) [11] and the European League Against Rheumatism (EULAR) have advocated for priority to be given to RMDs at the policy level, to reflect the significant challenges these conditions pose to public health. This review identifies some of the main challenges and opportunities for the RMDs community today (Fig. 2).

## Methodology

A preliminary PubMed literature search focusing on challenges in the areas of RMDs clinical care, education and research was conducted to identify key questions for the authors to address. Subsequently, a pre-agreed agenda was discussed at the inaugural meeting of the World Forum on Rheumatic and Musculoskeletal Diseases (WFRMD), held in Abu Dhabi on 26 September 2014. A more focused literature search was then conducted on the key topics identified, with further discussions between authors during development of the final manuscript.

## Challenges within the clinical care services

According to the WHO, a key component of a well-functioning health system is to provide equitable access to people-centred care [12]. Availability of healthcare workers, clinical services, affordability of care and cultural acceptability of treatment are all important factors [13]. Any disparity and inadequacy in patient access to healthcare professionals, including primary care providers (PCPs^2^) rheumatologists, orthopaedic surgeons, physical medicine and rehabilitation specialists, nurses, occupational therapists and physiotherapists can significantly delay diagnosis of RMDs and treatment initiation, both of which are key to minimising disease progression and improving patient outcomes [14, 15].

### Access to rheumatology services

Although the number of rheumatologists in developed countries far exceeds that in developing countries, there are worldwide (Table 1) and regional shortfalls in the provision of rheumatologists. A Markov prediction model examining supply and demand for rheumatologists in the United States (US) found that the situation is likely to worsen over the coming decades [16], largely due to an ageing population. Increasing rates of non-communicable diseases, on top of existing endemic and emerging diseases, are also likely to compete for limited resources [17].

**Table 1:** Estimated rheumatologist workforce across different countries

Country	Year data available	Population	Estimated number of rheumatologists	Ratio (per 100,000 population)
Europe				
UK [64]	2011	63,258,918	531^a^	0.84
Ireland [65]	2011	4,576,794	∼23	0.5^b^
France [66]	2010	65,023,142	2,470^c^	3.80
Germany [67]	2011	81,797,673	757^d^	0.93
Italy^e^	2011	60,782,668	800	1.3
North America				
USA^f^	2012	313,873,685	5602	1.78
Canada [68]	2013	35,158,304	342	0.97
Middle East				
Oman^g^	2014	3,632,444	20	0.55
UAE^g^	2014	9,346,129	40	0.43
Kuwait^g^	2014	3,368,572	30	0.89
Qatar^g^	2014	2,168,673	12	0.55
Saudi Arabia^g^	2014	28,828,870	120	0.42
Bahrain^g^	2014	1,332,171	4	0.30
Latin America				
Uruguay^f^	2012	3,395,253	105	3.09
Brazil^f^	2012	198,656,019	1543	0.78
Colombia^f^	2012	47,704,427	136	0.29
Nicaragua^f^	2012	5,991,733	4	0.07
Mexico^f^	2012	120,847,477	568	0.47
Asia				
China [69]	2007	1,317,885,000	2216	0.17
India^g^	2014	1,252,139,596	∼200	0.02
Pakistan [22]	2014	182,142,594	20	0.01
Thailand [42]	2014	67,010,502	150	0.22
Africa				
Comoros^g^	2014	734,917	0	0
Nigeria^g^	2014	173,615,345	22	0.01
Djibouti^g^	2014	872,932	0	0
Australia				
Australia^g^	2014	23,130,900	307	1.33

^a^Number of full-time equivalent rheumatology physicians, May 2011

^b^Number of practising rheumatology physicians per 100,000, May 2011

^c^Number of practising rheumatology specialists, 2010

^d^Number of rheumatology physicians (with specialist certificate), May 2011

^e^Data on file, Italian Society for Rheumatology

^f^Data from PANLAR National Workforce Survey, 2012

^g^Number of rheumatologists estimated based on personal communication with rheumatologists. Population data were obtained from World Bank mid-year estimates [70] based on year for which the number of rheumatologists were available; 2013 population data was used for 2014 data as 2014 population estimates were not yet available. Ratios were calculated based on number of rheumatologists divided by population estimates for that year

Analysis of the distribution of rheumatology practices across the US using the 2010 American College of Rheumatology (ACR) membership database found that of 3920 practising rheumatologists, 90 % worked in metropolitan regions, with only 3 % practising in micropolitan areas (populations <50,000) and 7 % practising in rural areas [18]. Similarly, in Canada and the Latin American and Caribbean region, rheumatologists are distributed mainly in large cities, leaving micropolitan and rural areas underserved. Findings from two studies in Ontario reported fewer rheumatologist visits for arthritis and inflammatory arthritis patients who lived in less populated areas with lower socioeconomic status [19, 20]. As a consequence of the worldwide shortage of rheumatologists, RMD patients are more likely to receive attention from a PCP, who may have no formal training in rheumatology [21, 22].

While shortages are documented in rheumatology care in general, paediatric rheumatology care is severely restricted. In Africa, there are only two paediatric rheumatologists [23], with an estimated 159–180 practising paediatric rheumatologists across the Latin American and Caribbean regions, 325 in the US and around 60 paediatric rheumatologists in the UK. In India, reports suggest that <50 % of the 1.3 million children with juvenile idiopathic arthritis (JIA) are diagnosed during the first year of their illness due to limited paediatric rheumatology care [24].

Pan American League Against Rheumatism (PANLAR) and National Rheumatology Societies in the Latin American and Caribbean Region recognise that the number of rheumatologists is insufficient to meet demands [17, 25]. The use of geospatial analysis could aid understanding of the access to healthcare and distribution of the RMDs in any predetermined geographic area [26]. A study of US Medicare patients found that increased driving distance to rheumatologists was associated with decreased odds of diagnosis with rheumatoid arthritis (RA) [27]. Providing up-to-date information about the local supply of rheumatologists, and additional funds for training programmes, could attract new rheumatologists to underserved regions through migration and expansion [18]. Nevertheless, in some countries, it may never be possible to train a sufficient number of rheumatologists. As such, PCPs may need to play a greater role in early rheumatological care and provide a more effective triage system. The early arthritis clinic model is gaining popularity, in which PCPs are trained to screen patients with RMDs and triage the most severe and urgent cases, such as primary inflammatory arthropathies [28]. The use of telephone and videoconference consultations may provide an effective means to improve access to rheumatology care in rural areas. This would allow for both direct patient care by rheumatologists and support of PCPs who can be educated, mentored and given diagnostic and management advice [9, 29–32].

### Access to diagnostics

Early diagnosis and treatment of RMDs in the ‘window of opportunity’ following the onset of symptoms are crucial to avoid long-term complications but requires specialist knowledge [33]. When appropriate treatment is started early, medical costs, disability and work limitations can be reduced [33].

Currently, there is no single preferred method of diagnosing RMDs, and tools used are dependent on the PCPs’ own knowledge [34]. Clinical diagnoses are typically supported by blood tests (e.g. erythrocyte sedimentation rate, C-reactive protein, anti-nuclear antibodies, rheumatoid factor) and imaging modalities. However, lack of expertise or funding and limited imaging facilities can create barriers to more systematic use of magnetic resonance imaging (MRI) and ultrasound in some regions, including Latin America [35]. Use of dual-energy X-ray absorptiometry (DXA) to measure bone mineral density is the standard diagnostic technique for osteoporosis but costs are relatively high, with limited access to equipment in many developing countries [36]. Absolute fracture risk calculators such as the FRAX model (a validated web-based algorithm) may provide a suitable alternative [37]. Common serological tests such as rheumatoid factor and anti-nuclear antibodies are available in all African countries, but rural areas invariably do not have such facilities. In resource-poor settings, with limited access to diagnostics, the assessment of disease activity using clinical rather than laboratory measures could be advantageous (e.g. the modified systemic lupus erythematosus [SLE] Disease Activity Index [SLEDAI]) [38].

Further development of rapid blood or serum-based diagnostic tests to screen for auto-immune RMDs should be encouraged. Point-of-care tests for early detection of RA using auto-antibodies or rheumatoid factor require only a single drop of blood and can be performed within minutes [39]. These advanced and non-invasive diagnostic tools represent an expanding area of interest for rheumatologists. Use of capillaroscopy to examine microcirculatory impairment is also gaining popularity amongst Western and Eastern countries, with dedicated study groups at ACR and EULAR congresses.

Musculoskeletal ultrasound (MSUS) is playing an increasingly important role in optimising clinical assessment of patients with RMDs and substantially improves therapeutic and diagnostic capabilities [40]. However, training is expensive, labour intensive and the learning curve is very steep [41], with huge variation between countries signifying the need for an international consensus on MSUS training [41]. ACR, EULAR and PANLAR have all developed guidelines and international training programmes for MSUS.

### Access to therapies

Access to suitable therapies is largely dependent on the nation’s health system, drug availability and the economic status of the country. In wealthy countries, there may be easy access to therapies through government-supported funding or insurance, whereas in poorer countries, patients are offered treatment solely based on their ability to pay for it. In Thailand, patients in the Universal Coverage Scheme can only access medicine on the National List of Essential Medicines, which does not include biologics, and treatment regimens are modified according to the level of patient health insurance cover [42]. Even in the US, patients with insurance coverage may be forced by the insurer to pay high co-payments or “co-insurance”. These additional payments may place vital treatment out of the patient’s grasp. The ACR spends great time and resources to combat these onerous practices in cooperation with its advocacy partner, the Arthritis Foundation. In Africa, immunosuppressants for the treatment of SLE are unaffordable for the majority of patients, or even state healthcare budgets [38]. Such variable access to therapies can deny implementation of established RMD treatment guidelines in developing countries, such as EULAR recommendations on the use of biologics in RA [43].

In 2011, a cross-sectional study across 46 European countries found that patients with RA in lower income countries had reduced access to both biologic and synthetic disease-modifying anti-rheumatic drugs (bDMARDs and sDMARDs) [13]. PANLAR has proposed that biosimilars could improve therapy access for patients with RA in the Latin American countries, as could decreasing custom fees and taxes for drugs and joint prostheses [25]. Informal discussions with manufacturers in the US indicates that the cost of biosimilars may not be significantly reduced compared to biologics, with ≤20 % price reduction anticipated. However, in the Norwegian tender system, the biosimilar Remsima was offered for a price 39 % lower than that for Remicade [44].

Inequities exist in the availability and consistency of supply of established cost-effective medications for RMDs, including non-steroidal anti-inflammatory drugs (NSAIDs; such as ibuprofen, naproxen and diclofenac) and sDMARDs, such as hydroxychloroquine, sulfasalazine, methotrexate (MTX) and leflunomide. In some African countries, disease-modifying anti-rheumatic drugs (DMARDs) are usually stocked by only a few pharmacies. The time taken for patients to receive sDMARDs may also vary depending on the healthcare system; in Brazil, it can take 1–4 years before a patient in the public healthcare system receives a sDMARD, compared with <2 years in private healthcare [35]. In South Sudan, DMARDs are not generally available and in other African countries, similar issues exist with SLE medications, alongside the fear of buying counterfeit medications locally [38]. Consequently, patients and their doctors need to obtain medicines from other countries, often at great expense [45, 38].

The concept of selling essential medicines in low- and middle-income countries at lower prices than in industrialised countries has received widespread support from industry, policy-makers and academics [46]. However, tiered pricing does not necessarily result in the lowest sustainable prices or lead to price reductions over time [46]. Further research is needed to determine whether a tiered pricing model could successfully increase access to RMD treatments in developing countries.

## Education of rheumatologists and other experts in RMD care

Successful education in the RMDs requires a collaborative, constructive, and contextual approach, aimed at providing education across professions. To address the current challenges in education across the medical education continuum, leadership of RMD experts specialising in medical education, research and service leadership will be mandatory.

### Medical school

Early exposure of medical students, residents and students of other relevant health professions to rheumatology ‘culture’ is vital to improve awareness of the RMDs and increase the number of students and residents considering this field of study. In many countries, PCPs lack knowledge of the nuances of RMDs, leading to misconceptions about the most appropriate treatment options, delayed diagnoses and a limited awareness of conditions such as the spondylarthropathies.

To date, rheumatology education has not been a high priority in many medical school curricula and consequently does not allow sufficient exposure of students to the RMDs [47]. A 2008 survey of Canadian medical schools found that the total average time spent in musculoskeletal physical examination teaching was just 4–7 h over 4 years of study, and 58 % of teaching was performed by non-RMD experts [48, 49]. In part, this may be due to the limited number of rheumatologists and other health professionals trained as educators and educational leaders, who can combine their expertise in both RMDs and education to increase focus on RMDs across the education continuum.

### Generalist training

A US survey of PCPs evaluated their experience with the use of DMARDs in RA and found that only a minority initiated this therapy option and PCPs generally had a high level of discomfort prescribing DMARDs [50]. A similar US survey to assess the use of European and American gout treatment recommendations found that of 838 PCP respondents, only half reported optimal treatment practices for the management of acute gout, and <20 % for intercritical or tophaceous gout, indicating care deficiencies [51]. Typically, PCPs are over-extended taking care of other chronic diseases, such as obesity, hypertension and diabetes, which may explain their restricted time devoted to increased understanding of RMDs.

In a survey of undergraduate nursing, occupational therapy and physiotherapy courses in the UK, educationalists reported only limited coverage of rheumatology [52, 53]. However, the ACR’s Association of Rheumatology Health Professionals (ARHP) offers a successful programme called Advanced Practice Rheumatology, with a modular online course and hands-on training for advanced practice nurses and physician’s assistants. Other societies have similar programmes such as the Rheumatology Nurses Society (RNS), committed to the education of nurses working in rheumatology. In the Latin American countries, a PANLAR-endorsed multinational rheumatology e-learning and presence training diploma programme for nurses and health professionals has been established and includes a hands-on and theoretical MSUS course. Greater provision of online training courses in developing countries, at reduced costs, could further enhance training opportunities.

Paediatric rheumatology is a particular area in which PCPs require further education. A review of the barriers constraining access to appropriate paediatric rheumatology care found that, in general, US paediatricians had little or no training in RMD health and had relatively poor physical examination and diagnosis skills [54]. Introduction of a mandatory RMDs rotation for paediatric residencies could help to improve awareness and understanding of these cases [54]. The ACR currently funds visiting paediatric rheumatology professorships, to bring in-depth training to paediatric programmes and provide much needed support for paediatric care in developing countries.

### Specialist training

Collaborations between rheumatology associations and universities could help strengthen existing teaching, provide core knowledge of the RMDs to all practitioners and encourage rheumatology as a specialty within that region. The ACR provides training and research grants for medical students, residents, fellows and clinicians in rheumatology, and has convened an international task force to assess the need for, and distribution of, educational tools. Similarly, the EULAR Committee for Education and Training (ESCET) offers bursaries for students and rheumatologists worldwide to attend online and postgraduate courses on RMDs. The Emerging EULAR Network (EMEUNET) is a working group of young rheumatology clinicians across 77 countries that facilitates education in RMDs. EULAR also offers a subsidised online rheumatology course.

Nevertheless, there is a general lack of training opportunities for those considering specialisation as rheumatologists. For example, in Nigeria, there is only one rheumatology training centre in the country. In Thailand, there are only 15 rheumatology training positions available each year, but this could be improved by increasing the number of grants available from the Thai Ministry of Public Health [42]. Of the current rheumatologists practising in India and Pakistan, 20 and 50 %, respectively, received their training elsewhere [22]. In the United Arab Emirates, Bahrain and Oman, all rheumatology training takes place overseas, while in Kuwait, Qatar and Saudi Arabia residents have hybrid programmes of local and international training. Although overseas accreditation can be beneficial, the majority who train in the West do not return to their native countries to practice rheumatology [22].

### Continuing education

Regional and national rheumatology leagues should help to deliver courses with clear curricular goals. The Chinese Rheumatology Association is working with medical schools to provide intensive training sessions, lectures and comprehensive curricula for the purpose of continuing medical education (CME) [55]. The ACR Rheumatology Research Foundation also has programmes for this, plus nascent programmes to attract college students to rheumatology and other rheumatology health professions.

Reduced registration fees or sponsorship to attend international congresses presents an ideal opportunity to promote ‘rheumatology without borders’ so that physicians worldwide can learn about the latest advances in the treatment of RMDs [56]. The impact of educational forums could be broadened via free post-congress web links that disseminate talks and workshops (using podcasts, videos, slide sets, handouts and translated materials) to target audiences that are unable to attend the congress due to lack of time or funding, travel requirements and language barriers. In addition, world rheumatology leaders could travel to developing regions to share their knowledge and expertise, e.g. the International Advanced Abu Dhabi Rheumatology Review Course (and numerous other meetings around the world).

CME programmes are currently offered by ACR, EULAR, American Board of Medical Specialties (ABMS), Royal College of Physicians (RCP), Asia Pacific League of Associations for Rheumatology (APLAR), PANLAR and African League against Rheumatism (AFLAR). However, there is a general lack of programmes in Africa, Asia and the Middle East. The International League of Associations for Rheumatology (ILAR) Grants Program also provides opportunities to advance the education and clinical practice of rheumatology in developing countries but has limited resources, with grants totalling $150,000 in 2014. A successful ILAR-supported project running from 2012 to 2013 addressed a particular need in Zambia to enhance paediatric and adult rheumatology education and practice [57]. Similarly, the ILAR-funded UWEZO Musculoskeletal Health training project aims to provide medics in Kenya with appropriate training to diagnose and treat RMDs (UWEZO means ‘capability’ in Swahili). Trained medics can then go on to train community health workers in their region.

The Arthritis Alliance of Canada has developed a coordinated national framework for models of care for patients with inflammatory joint diseases. Similarly, the Canadian Rheumatology Association (CRA) is a strong and committed organisation for RMDs, forming alliances with the Canadian Medical Association and the Royal College of Physicians and Surgeons, and increasing relationships with other organisations including ACR and PANLAR.

### Patient awareness of RMDs

In some areas of the world, a lack of basic education and literacy, poorer socioeconomic status and cultural beliefs can influence the decision of patients to seek medical attention from a PCP, or they may only seek help from alternate care practitioners [9]. Many patients see the aches and pains associated with arthritis as a part of life and do not seek medical opinion.

Prevention strategies with campaigns to educate and raise awareness about RMDs may increase the likelihood of a patient approaching their PCP and may also increase a patient’s understanding of their disease, ultimately improving their compliance with treatment decisions. The Patient Association inside EULAR (PARE) organises courses and local training sessions for patients across 37 countries. Existing campaigns to educate the public about health issues such as diabetes, hypertension and obesity could also be expanded to include RMDs. With the increasing use of smartphones worldwide, patient self-management or education programmes integrated into social media technologies or mobile applications might broaden patient access to information about their disease [58]. Looking ahead, organisations such as the US Arthritis Foundation or the ACR *Simple Tasks* campaign, which has gained the interest of rheumatology associations worldwide, may play an important role in raising patient and physician awareness and educating policy-makers about the value of rheumatology education [33].

## Research challenges

The impact of financial, political, social, or environmental factors on the opportunity to conduct clinical, epidemiological, as well as basic and translational research creates major challenges worldwide. In many countries, including the US and Canada, government funding for rheumatology research is diminishing and academics are being forced into the clinic, reducing their research time. In countries with major shortfalls in the rheumatology workforce, health systems are likely to need all rheumatologists for full-time clinical practice, leaving insufficient time for research activities.

### Clinical trials research

Ethnicity may influence the manifestation of various RMDs, due to underlying genetic differences, environmental factors, cultures and socioeconomic status [42]. However, pharmaceutical companies running randomised controlled trials (RCTs) have tended to conduct trials in Western industrialised countries, limiting the amount of information for the effectiveness and safety of treatments in non-Western populations (Table 2). Although more trials are now occurring in Eastern Europe and India due to a lack of biologic-naïve patients in the West, the Middle East and North Africa region sponsor less than 1 % of global clinical trials [59].

**Table 2:** Number of registered clinical trials listed on ClinicalTrials.Gov for different RMDs by region

Region	Number of registered trials			
	Rheumatoid arthritis	Osteoarthritis	Gout	Systemic lupus erythematosus
Africa	75	24	8	13
East Asia	253	144	13	86
Europe	678	530	31	104
Latin America^a^	243	65	9	59
Middle East	89	53	2	16
North America	693	821	85	238
North Asia	160	12	12	30
Pacifica	102	43	9	16
South Asia	48	16	2	16
Southeast Asia	61	39	8	24

Information based on number of clinical trials registered on ClinicalTrials.Gov by RMD topic, as of 4 November 2014. Studies with no locations are not included; studies with multiple locations are included in each region containing locations

^a^Latin America values obtained by adding number of trials in Central America and South America

Action is needed to encourage pharmaceutical companies to conduct RCTs which are more generalisable to different populations. However, a number of challenges must be overcome including training individuals to successfully manage the trials, differences in language, culture, social and health literacy; inaccurate translation and high levels of illiteracy can be problematic [59]. Establishing local Clinical Research Organisations could help overcome regulatory bottlenecks, as they are more likely to understand local regulations and logistic needs [59]. More discussion groups at rheumatology congresses such as ACR, EULAR, AFLAR and PANLAR on how to conduct RCTs may also help to identify potential issues for developing countries. In developed countries, increased dialogue is needed between drug regulatory bodies such as the US Food and Drug Administration (FDA) and European Medicines Agency (EMA), pharmaceutical companies and researchers coordinating RCTs. Recently, collaboration between EULAR and the EMA has been officialised, with EULAR members making recommendations together with EMA officers.

### Investigator-initiated research

There are many existing programmes to support investigator-initiated RMDs research including the Canadian Initiative for Outcomes in Rheumatology Care (CIORA) which offers awards to new clinicians to carry out independent arthritis research. Similarly, the ACR Rheumatology Research Foundation funds promising research to help bridge investigators to sustainable support from the National Institutes of Health (NIH). In China, government funding for rheumatology research has increased substantially in the past decade, leading to many high-quality publications and international collaborations [55]. In the recent Arab League Against Rheumatism (ARLAR) meeting, 100 research scholarships were awarded to young rheumatologists. The Paediatric Rheumatology International Trials Organisation (PRINTO), Paediatric Rheumatology European Society (PReS) and Childhood Arthritis and Rheumatology Research Alliance (CARRA) also aim to facilitate and coordinate clinical trials and research in paediatric RMDs.

### Epidemiological research

Real-life efficacy and safety data from registries provide a valuable source of data for the RMDs. For example, the British Society for Rheumatology Biologics Register (BSRBR) has been used to compare the risk of tuberculosis between different biologics used to treat RA [60]. The nascent ACR RISE registry will collect point-of-service data on patients in various settings, with international interest in joining this registry. Similarly, BIOBADAMERICA is a Latin America and Caribbean registry of 15 nations that collects information on all relevant adverse events in patients on biologics [61].

To date, the most comprehensive effort to collect data for the RMDs is the Global Burden of Disease (GBD) 2010 study, which estimated the global burden of RMDs and showed that the prevalence and burden of RMDs are exceptionally high throughout the world [2–7, 62]. However, for many regions, burden estimates had to be derived through predictive modelling [8].

Country-based data is very useful for local clinicians to follow their own patients who may be different in terms of disease severity, access to care, and comorbidities. Such data is also beneficial when negotiating with local governments and payers to provide evidence for the benefit and safety of more expensive treatments. Despite the obvious advantages of collating such information, obtaining the funding to set up new registries can be difficult. Furthermore, academics are not necessarily engaged in this type of activity. In many developing countries, health records are still paper-based; the introduction of electronic records is necessary to maximise data collection and simplify registry management.

The WHO-ILAR Community Oriented Programme in the Rheumatic Diseases (COPCORD) was created to gather data on RMDs, with an emphasis on developing rural economies [63]. The COPCORD Core Questionnaire (CCQ) has provided a useful way of collecting grass-roots data on the prevalence of RMDs in developing countries. However, the methodology is quite labour intensive, and design and methods are limited by stringent budgets, imposing deviations in the core protocol [63]. To harmonise epidemiological data for the RMDs, valid and standardised questions should be developed for use in population-based health interview and examination surveys.

## Conclusions

The high prevalence and burden of RMDs presents a number of global challenges for the practice of rheumatology and more generally for chronic diseases. Issues concerning access to clinical care services, rheumatology education and research are particularly problematic in developing countries and rural areas, yet challenges faced in developed countries hinder the progress of RMD healthcare worldwide. By increasing awareness of the burden of RMDs at the policy-maker level and identifying key challenges, it may be possible to identify realistic opportunities to address the global RMDs problem. The WFRMD aims to address important challenges for the RMDs by facilitating discussions with rheumatology experts and lobbying stakeholders, internal and regional rheumatology associations, local governments and the WHO to raise awareness of these prevalent and burdensome diseases.

## Electronic supplementary material

Below is the link to the electronic supplementary material.

ESM 1 (PDF 50 kb)

## Footnotes

1 For the purpose of this manuscript, developing countries are defined by World Bank criteria as low- and middle-income economies, i.e. those with gross national income per capita calculated using the World Bank Atlas method, of $1045–12,746 in 2013.

2 PCPs are healthcare practitioners who provide primary care for patients, defined by the WHO as first-contact, accessible, continued, comprehensive care, which coordinates with other specialists to provide any other care the patient may need.

## References

[1] Vos T, Flaxman AD, Naghavi M, Lozano R, Michaud C, Ezzati M, Shibuya K, Salomon JA, Abdalla S, Aboyans V (2013). Years lived with disability (YLDs) for 1160 sequelae of 289 diseases and injuries 1990–2010: a systematic analysis for the global burden of disease study 2010. Lancet.

[2] Cross M, Smith E, Hoy D, Carmona L, Wolfe F, Vos T, Williams B, Gabriel S, Lassere M, Johns N (2014). The global burden of rheumatoid arthritis: estimates from the global burden of disease 2010 study. Ann Rheum Dis.

[3] Cross M, Smith E, Hoy D, Nolte S, Ackerman I, Fransen M, Bridgett L, Williams S, Guillemin F, Hill CL (2014). The global burden of hip and knee osteoarthritis: estimates from the global burden of disease 2010 study. Ann Rheum Dis.

[4] Hoy D, March L, Brooks P, Blyth F, Woolf A, Bain C, Williams G, Smith E, Vos T, Barendregt J (2014). The global burden of low back pain: estimates from the global burden of disease 2010 study. Ann Rheum Dis.

[5] Hoy D, March L, Woolf A, Blyth F, Brooks P, Smith E, Vos T, Barendregt J, Blore J, Murray C, Burstein R, Buchbinder R (2014). The global burden of neck pain: estimates from the global burden of disease 2010 study. Ann Rheum Dis.

[6] Smith E, Hoy D, Cross M, Merriman TR, Vos T, Buchbinder R, Woolf A, March L (2014). The global burden of gout: estimates from the global burden of disease 2010 study. Ann Rheum Dis.

[7] Smith E, Hoy DG, Cross M, Vos T, Naghavi M, Buchbinder R, Woolf AD, March L (2014). The global burden of other musculoskeletal disorders: estimates from the global burden of disease 2010 study. Ann Rheum Dis.

[8] Hoy DG, Smith E, Cross M, Sanchez-Riera L, Blyth FM, Buchbinder R, Woolf AD, Driscoll T, Brooks P, March LM (2014) Reflecting on the global burden of musculoskeletal conditions: lessons learnt from the global burden of disease 2010 study and the next steps forward. Ann. Rheum Dis:annrheumdis-2014-205393 10.1136/annrheumdis-2014-205393 24914071

[9] Mody GM, Brooks PM (2012). Improving musculoskeletal health: global issues. Best Pract Res Clin Rheumatol.

[10] Massardo L, Pons‐Estel BA, Wojdyla D, Cardiel MH, Galarza-Maldonado CM, Sacnun MP, Soriano ER, Laurindo IM, Acevedo-Vásquez EM, Caballero-Uribe CV (2012). Early rheumatoid arthritis in Latin America: low socioeconomic status related to high disease activity at baseline. Arthritis Care Res.

[11] Woolf AD The bone and joint decade: working together to make musculoskeletal conditions a public health priority. http://www.arthritisresearchuk.org/health-professionals-and-students/reports/topical-reviews/topical-reviews-summer-2012.aspx#sthash.AvOx0hMb.dpuf. Accessed 5th Nov 2014

[12] Key components of a well functioning health system. World Health Organization, 2010. http://www.whoint/healthsystems/EN_HSSkeycomponentspdf?ua=1 Accessed 5th Nov 2014

[13] Putrik P, Ramiro S, Kvien TK, Sokka T, Pavlova M, Uhlig T, Boonen A, Tafaj A, Harutyunyan R, Radner H (2014). Inequities in access to biologic and synthetic DMARDs across 46 European countries. Ann Rheum Dis.

[14] Fiehn C, Hajjar Y, Mueller K, Waldherr R, Ho A, Andrassy K (2003). Improved clinical outcome of lupus nephritis during the past decade: importance of early diagnosis and treatment. Ann Rheum Dis.

[15] Nell V, Machold K, Eberl G, Stamm T, Uffmann M, Smolen J (2004). Benefit of very early referral and very early therapy with disease-modifying anti-rheumatic drugs in patients with early rheumatoid arthritis. Rheumatology.

[16] Deal CL, Hooker R, Harrington T, Birnbaum N, Hogan P, Bouchery E, Klein‐Gitelman M, Barr W (2007). The United States rheumatology workforce: supply and demand, 2005–2025. Arthritis Rheum.

[17] Cardiel MH (2011). Present and future of rheumatic diseases in Latin America. Are we prepared to face them?. Reumatología Clínica (Engl Ed).

[18] FitzGerald JD, Battistone M, Brown CR, Cannella AC, Chakravarty E, Gelber AC, Lozada CJ, Punaro M, Slusher B, Abelson A (2013). Regional distribution of adult rheumatologists. Arthritis Rheuma.

[19] Badley EM, Canizares M, Gunz AC, Davis AM (2014) Visits to rheumatologists for arthritis: the role of access to primary care physicians, geographic availability of rheumatologists, and socioeconomic status. Arthritis Care Res 10.1002/acr.22413 25048206

[20] Widdifield J, Paterson JM, Bernatsky S, Tu K, Thorne JC, Ivers N, Butt D, Jaakkimainen RL, Gunraj N, Ahluwalia V (2014). Access to rheumatologists among patients with newly diagnosed rheumatoid arthritis in a Canadian universal public healthcare system. BMJ Open.

[21] Oyoo O, Moots RJ, Ganda B (2012) Stepping into the state of rheumatology in East Africa. Rheumatology:ker411 10.1093/rheumatology/ker411 22319079

[22] Gibson T (2014) Rheumatology in India and Pakistan today. Rheumatology:keu306 10.1093/rheumatology/keu306 25102860

[23] Henrickson M (2011). Policy challenges for the pediatric rheumatology workforce: Part III. The international situation. Pediatr Rheumatol Online J.

[24] Habibi S, Aggarwal A, Ramanan AV (2012). Paediatric rheumatology in India: challenges and opportunities. Rheumatology.

[25] Cardiel M (2006). First Latin American position paper on the pharmacological treatment of rheumatoid arthritis. Rheumatology.

[26] Al‐Maini M, Jeyalingam T, Brown P, Lee JJ, Li L, Su J, Gladman DD, Fortin PR (2013). A hot spot for systemic lupus erythematosus, but not for psoriatic arthritis, identified by spatial analysis suggests an interaction between ethnicity and place of residence. Arthritis Rheum.

[27] Polinski JM, Brookhart MA, Ayanian JZ, Katz JN, Kim SC, Lii J, Tonner C, Yelin E, Solomon DH (2014) Relationships between driving distance, rheumatoid arthritis diagnosis, and disease‐modifying anti‐rheumatic drug receipt. Arthritis Care Res 10.1002/acr.22333 PMC4175286 24664991

[28] Gormley GJ, Steele WK, Gilliland A, Leggett P, Wright GD, Bell AL, Matthews C, Meenagh G, Wylie E, Mulligan R, Stevenson M, O’Reilly D, Taggart AJ (2003). Can diagnostic triage by general practitioners or rheumatology nurses improve the positive predictive value of referrals to early arthritis clinics?. Rheumatology (Oxford).

[29] Davis P, Howard R, Brockway P (2001). An evaluation of telehealth in the provision of rheumatologic consults to a remote area. J Rheumatol.

[30] Jong M, Kraishi M (2004). A comparative study on the utility of telehealth in the provision of rheumatology services to rural and northern communities. Int J Circumpolar Health.

[31] Leggett P, Graham L, Steele K, Gilliland A, Stevenson M, O’Reilly D, Wootton R, Taggart A (2001). Telerheumatology—diagnostic accuracy and acceptability to patient, specialist, and general practitioner. Br J Gen Pract.

[32] Roberts L, Lamont E, Lim I, Sabesan S, Barrett C (2012). Telerheumatology: an idea whose time has come. Intern Med J.

[33] ACR Simple Tasks Campaign Fact Sheet. http://simpletasks.org/wp-content/uploads/2013/12/Campaign-Fact_Sheet.pdf. Accessed 5th Nov 2014

[34] National Audit Office (NAO) (2009). Services for people with rheumatoid arthritis. Published by the NAO, London, 15 July 2009. http://www.nao.org.uk/publications/0809/rheumatoid_arthritis.aspx. Accessed 5th Nov 2014

[35] Miltenburger C, Munkombwe M, I L A Survey of Barriers to Treatment Access in Rheumatoid Arthritis in Major Latin American Countries—Argentina, Brazil and Mexico. March 2010. http://www.comparatorreports.se/LA%20RA%20barrier%20report_FINAL.pdf. Accessed 5th Nov 2014

[36] Kruger MC, Todd JM, Schollum LM, Kuhn-Sherlock B, McLean DW, Wylie K (2013). Bone health comparison in seven Asian countries using calcaneal ultrasound. BMC Musculoskelet Disord.

[37] Kanis JA, Johnell O, Oden A, Johansson H, McCloskey E (2008). FRAX and the assessment of fracture probability in men and women from the UK. Osteoporos Int.

[38] Tiffin N, Hodkinson B, Okpechi I (2013) Lupus in Africa: can we dispel the myths and face the challenges? Lupus:0961203313509296 10.1177/0961203313509296 24174511

[39] Egerer K, Feist E, Burmester G-R (2009). The serological diagnosis of rheumatoid arthritis. Deutsches Aerzteblatt.

[40] Naredo E, Iagnocco A (2012). Why use ultrasound in rheumatology?. Rheumatology.

[41] Taggart A, Benson C, Kane D Ultrasound in rheumatology

[42] Louthrenoo W (2014) An insight into rheumatology in Thailand. Nat Rev Rheumatol. doi:101038/nrrheum2014142 10.1038/nrrheum.2014.142 25201382

[43] El Zorkany B, AlWahshi HA, Hammoudeh M, Al Emadi S, Benitha R, Al Awadhi A, Bouajina E, Laatar A, El Badawy S, Al Badi M (2013). Suboptimal management of rheumatoid arthritis in the Middle East and Africa: could the EULAR recommendations be the start of a solution?. Clin Rheumatol.

[44] Moum B, Lundin KE (2014). Biosimilar medicines in inflammatory bowel disease. Tidsskr Nor Laegeforen Tidsskr Prakt Med Ny Raekke.

[45] Radis C (2012). Rheumatoid arthritis: diagnosis and treatment with a particular emphasis on South Sudan. S Sudan Med J.

[46] Moon S, Jambert E, Childs M, von Schoen-Angerer T (2011). A ‘win-win solution?’: A critical analysis of tiered pricing to improve access to medicines in developing countries. Glob Health.

[47] Freedman KB, Bernstein J (2002). Educational deficiencies in musculoskeletal medicine. J Bone Joint Surg.

[48] Oswald AE, Bell MJ, Snell L, Wiseman J (2008). The current state of musculoskeletal clinical skills teaching for preclerkship medical students. J Rheumatol.

[49] Thompson AE (2008). Improving undergraduate musculoskeletal education: a continuing challenge. J Rheumatol.

[50] Garneau KL, Iversen MD, Tsao H, Solomon DH (2011). Primary care physicians’ perspectives towards managing rheumatoid arthritis: room for improvement. Arthritis Res Ther.

[51] Harrold LR, Mazor KM, Negron A, Ogarek J, Firneno C, Yood RA (2013). Primary care providers’ knowledge, beliefs and treatment practices for gout: results of a physician questionnaire. Rheumatology.

[52] Almeida C, Clarke B, O’Brien A, Hammond A, Ryan S, Kay L, Hewlett S (2006). Current provision of rheumatology education for undergraduate nursing, occupational therapy and physiotherapy students in the UK. Rheumatology.

[53] Hewlett S, Clarke B, O’Brien A, Hammond A, Ryan S, Kay L, Richards P, Almeida C (2008). Rheumatology education for undergraduate nursing, physiotherapy and occupational therapy students in the UK: standards, challenges and solutions. Rheumatology.

[54] Henrickson M (2011). Policy challenges for the pediatric rheumatology workforce: Part I. Education and economics. Pediatr rheumatol Online J.

[55] Li Z, Yang Y (2012). Rheumatology in China: challenges and development. Rheumatology.

[56] Lau C, Feng P (2007). Rheumatology without borders. Nat Clin Pract Rheumatol.

[57] Chipeta J, Njobvu P, McGill PE, Bucala R (2014) Progress made towards enhancement of rheumatology education and practice in Zambia: review of an ILAR-supported project. Clin Rheumatol 1–6 10.1007/s10067-014-2624-0 PMC4161929 24752350

[58] Berenbaum F (2014). The social (media) side to rheumatology. Nat Rev Rheumatol.

[59] Nair SC, Ibrahim H, Celentano DD (2013). Clinical trials in the Middle East and North Africa (MENA) region: grandstanding or grandeur?. Contemp Clin Trials.

[60] Solovic I, Sester M, Gomez-Reino J, Rieder H, Ehlers S, Milburn H, Kampmann B, Hellmich B, Groves R, Schreiber S (2010). The risk of tuberculosis related to tumour necrosis factor antagonist therapies: a TBNET consensus statement. Eur Respir J.

[61] Registro Panamericano de Acontecimientos Adversos de terapias biológicas en enfermedades reumáticas. BIOBADAMERICA Website. https://biobadaser.ser.es/biobadamerica/. Accessed 5th November 2014

[62] Sanchez-Riera L, Carnahan E, Vos T, Veerman L, Norman R, Lim S, Hoy D, Smith E, Wilson N, Nolla J (2014). The global burden attributable to low bone mineral density. Ann Rheum Dis.

[63] Chopra A (2013). The COPCORD world of musculoskeletal pain and arthritis. Rheumatology.

[64] eumusc.net. Musculoskeletal health key statistics, United Kingdom. http://www.eumusc.net/map/fs_pdf/FS_uk.pdf. Accessed 5th November 2014

[65] eumusc.net. Musculoskeletal health key statistics, Ireland. http://www.eumusc.net/map/fs_pdf/FS_ie.pdf. Accessed 5th November 2014

[66] eumusc.net. Musculoskeletal health key statistics, France. http://www.eumusc.net/map/fs_pdf/FS_fr.pdf. Accessed 5th November 2014

[67] eumusc.net. Musculoskeletal health key statistics, Germany. http://www.eumusc.net/map/fs_pdf/FS_de.pdf. Accessed 5th November 2014

[68] National Physician Survey, 2013. College of Family Physicians of Canada, Canadian Medical Association, Royal College of Physicians and Surgeons of Canada. http://nationalphysiciansurvey.ca/wp-content/uploads/2013/10/NPS-2013-Internal-Medicine-RRENr.pdf. Accessed 5th November 2014

[69] Zhang F (2009). The China rheumatology workforce: a status report. Int J Rheum Dis.

[70] The World Bank, total population data by country. http://data.worldbank.org/indicator/SP.POP.TOTL. Accessed 5th November 2014

