# Supplementary material for: The global challenges and opportunities in the practice of rheumatology: White paper by the World Forum on Rheumatic and Musculoskeletal Diseases
Source: Clin Rheumatol. 2014 Dec 14;34(5):819–29. doi: 10.1007/s10067-014-2841-6 (PMC4408363; doi:10.1007/s10067-014-2841-6)
Supplement: Supplementary file 1 — (PDF 50 kb) [file 10067_2014_2841_MOESM1_ESM.pdf]

**Article Title:** The Global Challenges and Opportunities in the Practice of Rheumatology: White Paper by the World Forum on Rheumatic and Musculoskeletal Diseases

**Journal Name:** Clinical Rheumatology

**Authors:** Mustafa Al Maini,[1] Femi Adelowo, Jamal Al Saleh, Yousef Al Weshahi, Gerd-Rüdiger Burmester, Maurizio Cutolo, Joseph Flood, Lyn March, Heather McDonald-Blumer, Kevin Pile, Carlos Pineda, Carter Thorne, Tore K Kvien

[1]Mafraq Hospital, Abu Dhabi, United Arab Emirates;

**Email address for corresponding author:** mmaini@wfrmd.org

# SUPPLEMENTARY MATERIAL

## Useful online resources for the Rheumatic and Musculoskeletal Diseases (RMDs)

### GLOBAL WEBSITES

- **World Forum for the Rheumatic and Musculoskeletal Diseases**
  http://www.wfrmd.org/
- **International League of Associations for Rheumatology (ILAR)**
  http://www.ilar.org/
- **Community Oriented Program for Control of Rheumatic Diseases (COPCORD)**
  http://www.copcord.org/
- **Pediatric Rheumatology INternational Trials Organisation (PRINTO)**
  https://www.printo.it/
- **United Nations Dag Hammarskjöld Library, Non-communicable diseases (NCDs) and the post-2015 development agenda**
  http://research.un.org/en/NCD2015
- **Rheumatology Nurses Society**
  http://rnsnurse.org/
- **Applied Clinical Research, Clinical Trials and Regulatory Affairs Journal**
  http://benthamscience.com/journal/index.php?journalID=acctra
- **FRAX® WHO Fracture Risk Assessment Tool**
  http://www.shef.ac.uk/FRAX/

## REGIONAL WEBSITES

***US***

- **American College of Rheumatology (ACR)**
  http://www.rheumatology.org/
  - **ACR RhMSUS program**
    http://www.rheumatology.org/RhMSUS/
  - **ACR Simple Tasks Campaign**
    http://simpletasks.org/about/
  - **ACR US Rheumatology Research Foundation**
    http://www.rheumatology.org/Foundation/
- **US Arthritis Foundation**
  http://www.arthritis.org/

***Canada***

- **Arthritis Alliance Canada**
  http://www.arthritisalliance.ca/en/
- **Canadian Rheumatology Association**
  http://rheum.ca/
  - **Canadian Initiative for Outcomes in Rheumatology cAre (CIORA)**
    http://rheum.ca/en/ciora/
- **Grand challenges Canada**
  http://www.grandchallenges.ca/

***Europe***

- **European League Against Rheumatism (EULAR)**
  http://www.eular.org/
  - **Emerging EULAR network (EMEUNET)**
    emeunet.eular.org
  - **EULAR online course on Rheumatic Diseases**
    http://www.eular.org/index.cfm?framePage=/edu_online_course.cfm
  - **National organisations of people with arthritis/rheumatism (PARE)**
    http://www.eular.org/
  - **EULAR Standing Committee on Education and Training (ESCET)**
    http://www.eular.org/
  - **EULAR Horizon 2020 Press Release**
    http://www.eular.org/congresspressreleases/Horizon_2020_and_FOREUM_FINAL.pdf
- **Eumusc.net**, **The European musculoskeletal surveillance and information network**

http://www.eumusc.net/

- **Pediatric Rheumatology European Society (PReS)**
  www.pres.org.uk

***Rest of world***

- **Pan-American League of Associations for Rheumatology (PANLAR)**
  http://www.panlar.org/
- **Asia Pacific League of Associations for Rheumatology (APLAR)**
  http://www.aplar.org/Pages/Home.aspx
- **Indian Rheumatology Association**
  http://www.indianrheumatology.org/
- **African League of Associations for Rheumatology (AFLAR)**
  http://www.aflar.net/index.php
- **Arab League Against Rheumatism (ARLAR)**
  http://www.arlar.org/
- **Abu Dhabi Advanced Rheumatology Review Course**
  http://www.adarrc.org/
